# County-Level Disparities in Heat-Related Emergencies

**DOI:** 10.1001/jamanetworkopen.2024.2845

**Published:** 2024-03-19

**Authors:** Tarun Ramesh, Gregory D. Wozniak, Hao Yu

**Affiliations:** 1 Department of Population Medicine, Harvard Medical School and Harvard Pilgrim Health Care Institute, Boston, Massachusetts; 2 American Medical Association, Chicago, Illinois

## Abstract

This cross-sectional study examines the distribution of emergency medical service activation across US countries during the heat wave in July 2023.

## Introduction

Climate change has led to the highest temperatures on record with increasing frequency and intensity of heat waves globally. Prior studies have highlighted the role of extreme heat waves on health and health care outcomes, such as cardiovascular^1^ and all-cause mortality^2^ as well as hospital admissions for electrolyte disorders, heat stroke, sepsis, and kidney failure during heat waves.^3^ The burden of heat-related emergencies has been established at the city level^4^ but remains unclear at the national level, which can better shape federal policy priorities. This study examined the distribution of emergency medical service (EMS) activation across all US counties during the most recent heat wave in July 2023 and identified characteristics of counties with high burden of EMS activation.

## Methods

This retrospective cross-sectional study used July 2023 heat-related EMS activation data from the Department of Health and Human Services Office of Climate Change and Health Equity (OCCHE) and monthly county temperature and precipitation from the US Centers for Disease Control and Prevention (CDC) Heat and Health tracker. EMS activations for heat-related emergencies were extracted from EMS patient care reports from the National Emergency Medical Services Information System Technical Assistance Center data collection, which represents more than 90% of all patient care reports. This study was determined as not human participant research by the Harvard Pilgrim Health Care Institute institutional review board; informed consent was waived because our data were publicly available and deidentified. The study followed STROBE reporting guidelines.

We first used bivariate analyses to describe the distribution of heat-related EMS activations before performing multivariable logistic regressions on whether a county had substantially high heat-related EMS activation, defined by OCCHE as greater than 200% of the national average (3.6 per 100 000 residents). Four multivariable logistic regression models were estimated. All included maximum county temperature and monthly precipitation in inches, but differed by additional variables, with models 1 and 2 including the CDC Social Vulnerability Index (SVI) and corresponding SVI quintiles and models 3 and 4 including the University of Wisconsin’s Area Deprivation Index (ADI) and corresponding quintiles (eAppendix in Supplement 1). All tests were 2-sided with an α level of .05 and conducted in Stata version 18.0 (StataCorp).

## Results

Of 3089 counties analyzed, 728 (23.6%) were found to have substantially high heat-related EMS activation. Our bivariate analyses showed that these counties were in areas with higher SVI and ADI, located predominately in the South, Midwest, and Southwest (Figure, A and B).

**Figure.**
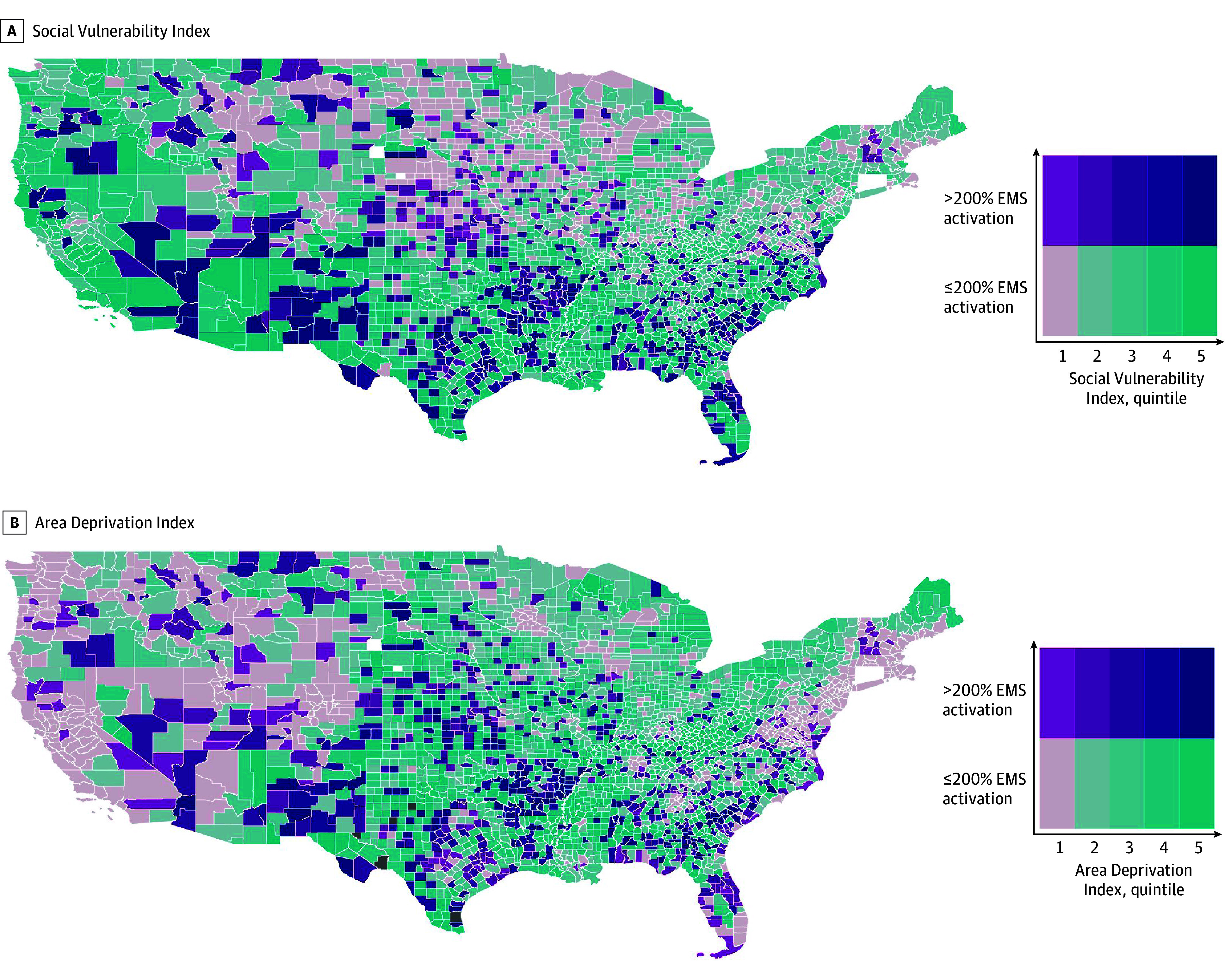
Counties by Emergency Medical Services (EMS) Activation for Heat-Related Emergencies and the Social Vulnerability Index and Area Deprivation Index Data from Alaska and Hawaii were not included in the maximum county temperature data and therefore not included in analysis. Substantially high EMS activation was defined by the US Department of Health and Human Services Office of Climate Change and Health Equity as greater than 200% of the national average. A, This map shows counties by EMS activation and Social Vulnerability Index quintiles with 5 being the highest quintile (ie, most vulnerable counties). Counties with both high EMS activation and social vulnerability were clustered in the South and Southwest. B, This map shows counties by EMS activation and Area Deprivation Index quintiles with 5 being the highest quintile (ie, most disadvantaged counties). Counties with both high EMS activation and area deprivation were clustered in the South, Midwest, and Southwest.

The estimates from our logistic regressions indicated that counties having substantially high heat-related EMS activation were more likely to have higher SVI (adjusted odds ratio [AOR], 1.77; 95% CI, 1.20-2.61) and higher ADI (AOR, 1.01; 95% CI, 1.00-1.02) when controlling for maximum county temperature and monthly precipitation. In the quintile analyses, counties with substantially high heat-related EMS activation were in counties in the highest SVI quintile (quintile 5: AOR, 1.64; 95% CI, 1.14-2.37) and counties in higher ADI quintiles (Table).

**Table.** Social Vulnerability Index and Area Deprivation Index for Counties With Substantially High Emergency Medical Services Activation for Heat-Related Emergencies

Model^a^	AOR (95% CI)	*P* value
Model 1		
Maximum temperature in July 2023	1.06 (1.03 - 1.09)	<.001
Monthly precipitation in July 2023	1.03 (0.98-1.07)	.22
Social Vulnerability Index	1.77 (1.20-2.61)	.004
Model 2		
Maximum temperature in July 2023	1.06 (1.04-1.09)	<.001
Monthly precipitation in July 2023	1.03 (0.98-1.07)	.20
Social Vulnerability Index		
Quintile 1	1 [Reference]	NA
Quintile 2	1.30 (0.89-1.90)	.18
Quintile 3	0.97 (0.66-1.41)	.86
Quintile 4	1.40 (0.97-2.03)	.08
Quintile 5	1.64 (1.14 - 2.37)	.008
Model 3		
Maximum temperature in July 2023	1.06 (1.04-1.09)	<.001
Monthly precipitation in July 2023	1.01 (0.97- 1.06)	.67
Area Deprivation Index	1.01 (1.00-1.02)	.001
Model 4		
Maximum temperature in July 2023	1.07 (1.05-1.10)	<.001
Monthly precipitation in July 2023	1.01 (0.97-1.06)	.60
Area Deprivation Index		
Quintile 1	1 [Reference]	NA
Quintile 2	1.32 (0.93-1.88)	.13
Quintile 3	1.82 (1.29-2.55)	.001
Quintile 4	1.75 (1.25-2.44)	.001
Quintile 5	1.49 (1.07-2.07)	.02

Abbreviations: AOR, adjusted odds ratio; NA, not applicable.

^a^ Models 1 and 2 include the Social Vulnerability Index and social vulnerability quintiles, respectively, with quintile 5 being the highest or most vulnerable counties. Models 3 and 4 include the Area Deprivation Index and area deprivation quintiles, respectively, with quintile 5 being the highest or most deprived counties. All models controlled for the county-level maximum temperature and monthly precipitation in inches in July 2023. Data from Alaska and Hawaii were not included in the US Centers for Disease Control and Prevention maximum county temperature data and therefore not included in analysis. Substantially high emergency medical service activation was defined by the Department of Health and Human Services Office of Climate Change and Health Equity as greater than 200% of the national average.

## Discussion

This study suggests that heat-related emergencies were more extensive in socially vulnerable and disadvantaged communities. These communities are already at greater risk for cardiovascular disease,^5^ with lower access to primary care^6^ compared with other counties.

Investment should target these communities to improve heat resilience. Our study limitations include county-level data without individual-level information and with temperature rather than heat index. Additionally, EMS activation represents one element of heat-related illness burden, with hospitalizations and mortality as critical aspects for further research. Additional research should assess the impacts of recent federal policy responses to extreme heat, such as the Low-Income Home Energy Assistance Program and the Building Resilient Infrastructure and Communities program, on individual health outcomes, especially in disadvantaged communities. Future studies should also evaluate within-county variation to better describe community health needs for heat-related emergencies.

## Notes

Supplement 1. **eAppendix.** Additional Information on SVI and ADI
eReferences.

Supplement 2.
Data Sharing Statement
