# Supplementary material for: County-Level Disparities in Heat-Related Emergencies
Source: JAMA Netw Open. 2024 Mar 19;7(3):e242845. doi: 10.1001/jamanetworkopen.2024.2845 (PMC10951733; doi:10.1001/jamanetworkopen.2024.2845)
Supplement: Supplement 1. — eAppendix. Additional Information on SVI and ADI eReferences. [file jamanetwopen-e242845-s001.pdf]

Supplemental Online Content

Ramesh T, Wozniak GD, Yu H. County-level disparities in heat-related emergencies. *JAMA Netw Open.* 2024;7(3):e242845. doi:10.1001/jamanetworkopen.2024.2845

eAppendix. Additional Information on SVI and ADI

eReferences.

This supplemental material has been provided by the authors to give readers additional information about their work.

© 2024 Ramesh T et al. *JAMA Network Open.*

eAppendix. Additional Information on SVI and ADI

The CDC's Social Vulnerability Index (SVI)[1] is a composite measure that includes 16 census variables which can affect a community's ability to prevent and respond to anthropogenic or natural disasters. It includes the following variables: Socioeconomic Status including below 150% Poverty, Unemployed, Housing Cost Burden, No High School Diploma, No Health Insurance; Household Characteristics including Aged 65 & Older, Aged 17 & Younger Civilian with a Disability, Single-Parent Households, English Language Proficiency, Racial & Ethnic Minority Status Hispanic or Latino (of any race); Black and African American, Not Hispanic or Latino; American Indian and Alaska Native, Not Hispanic or Latino; Asian, Not Hispanic or Latino; Native Hawaiian and Other Pacific Islander, Not Hispanic or Latino; Two or More Races, Not Hispanic or Latino; Other Races, Not Hispanic or Latino; Housing Type & Transportation including Multi-Unit Structures, Mobile Homes, Crowding, No Vehicle, and Group Quarters.

The Area Deprivation Index (ADI)[2] is a composite measure from the University of Wisconsin that includes census variables and other information to better understand neighborhood level disadvantage. It has been used in previous studies on breast cancer disparities[3] to advance health equity.[4]

© 2024 Ramesh T et al. *JAMA Network Open.*

eReferences.

1. CDC SVI Documentation 2020. Centers for Disease Control and Prevention. Available at: https://www.atsdr.cdc.gov/placeandhealth/svi/documentation/SVI_documentation_2020.html
2. Kind AJH, Buckingham W. Making Neighborhood Disadvantage Metrics Accessible: The Neighborhood Atlas. New England Journal of Medicine, 2018. 378: 2456-2458. DOI: 10.1056/NEJMp1802313. PMCID: PMC6051533. AND University of Wisconsin School of Medicine Public Health. 2015 Area Deprivation Index v2.0. Downloaded from https://www.neighborhoodatlas.medicine.wisc.edu/ May 23, 2019.
3. Luningham JM, Seth G, Saini G, et al. Association of Race and Area Deprivation With Breast Cancer Survival Among Black and White Women in the State of Georgia. JAMA Netw Open. 2022;5(10):e2238183. doi:10.1001/jamanetworkopen.2022.38183
4. "The Area Deprivation Index Is The Most Scientifically Validated Social Exposome Tool Available For Policies Advancing Health Equity", Health Affairs Forefront, July 20, 2023. DOI: 10.1377/forefront.20230714.676093

© 2024 Ramesh T et al. *JAMA Network Open.*
