# Supplementary material for: County-Level Disparities in Heat-Related Emergencies
Source: JAMA Netw Open. 2024 Mar 19;7(3):e242845. doi: 10.1001/jamanetworkopen.2024.2845 (PMC10951733; doi:10.1001/jamanetworkopen.2024.2845)
Supplement: Supplement 2. — Data Sharing Statement [file jamanetwopen-e242845-s002.pdf]

Data Sharing Statement

Ramesh. County-Level Disparities in Heat-Related Emergencies. *JAMA Netw Open*. Published March 19, 2024. doi:10.1001/jamanetworkopen.2024.2845

Data
Data available: Yes
Data types: Data (not involving human participants)
How to access data: The heat-related EMS activation data from the DHHS Office of Climate Change and Health Equity are publicly available at https://storymaps.arcgis.com/stories/93ea47545cc944139e3fcefa919cb42b. The monthly maximum county temperature from the CDC Heat and Health tracker is publicly available at https://ephtracking.cdc.gov/Applications/heatTracker/.
When available: With publication

Supporting Documents
Document types: None

Additional Information
Who can access the data: Any researchers who are interested in using the data.
Types of analyses: For research purpose.
Mechanisms of data availability: The data are publicly available.
Any additional restrictions: None.
